# Predictive Validity of a Functional Movement Screen in Professional Basketball Players

**DOI:** 10.3390/medicina56120724

**Published:** 2020-12-21

**Authors:** Donald L. Hoover, Clyde B. Killian, Rachel A. Tinius, David M. Bellar, Steven G. Wilkinson, Francis T. Esslinger, Lawrence W. Judge

**Affiliations:** 1 Doctor of Physical Therapy Department, Western Michigan University, Kalamazoo, MI 49008, USA; 2 Krannert School of Physical Therapy, University of Indianapolis, Indianapolis, IN 42667, USA; killianclyde@gmail.com; 3 School of Kinesiology, Recreation, and Sport, Western Michigan University, Bowling Green, KY 42101, USA; rachel.tinius@wku.edu (R.A.T.); francis.esslinger@wku.edu (F.T.E.); 4 Department of Kinesiology, University of North Carolina at Charlotte, Charlotte, NC 28223, USA; dbellar@uncc.edu; 5 Doctor of Physical Therapy Program, Rocky Mountain University of Health Professions, Provo, UT 84606, USA; steve.wilkinson@rm.edu; 6 School of Kinesiology, Ball State University, Muncie, IN 47306, USA; lwjudge@bsu.edu

**Keywords:** injury prevention, injury prediction, basketball injuries, non-contact injury, professional basketball

## Abstract

*Background and objectives*: Striking a balance between maximizing performance and preventing injury remains elusive in many professional sports. The purpose of this study was to assess the relative risk of non-contact injuries in professional basketball players based on predictive cut scores on the Functional Movement Screen™ (FMS). *Materials and Methods*: Thirty-two professional basketball players from the National Basketball Association (NBA) and Women’s National Basketball Association (WNBA) participated in this study. This observational pilot cohort study assessed and scored each participant using the FMS during training camp. Each athlete was then tracked throughout the season while recording the number, type, and time lost due to injuries. Possible exposures, actual exposures, and exposures missed due to non-contact injury (NCI) for each athlete were calculated and then used to determine the crude and specific incident rates for exposures missed due to NCI per 1000 exposures. *Results*: Linear regression models were used to evaluate the predictive ability of the FMS score for total missed exposures, NCI, and CI missed exposures. In all models, the FMS total score failed to attain significance as a predictor (*p* > 0.05). FMS scores ranged from 5 to 18. The recommended cut score of 14 showed a sensitivity of 0.474 and a specificity of 0.750. The cut score of 15 showed the best combination, exhibiting a sensitivity of 0.579 and specificity of 0.625. A total of 5784 exposures to NCI were possible for the men and women combined, and 681 possible exposures were missed. Of these, 23.5% were due to NCI, 16.5% were due to contact injuries (CI), and 60% were due to illnesses and personal reasons. *Conclusions*: The FMS proved to be a measure that was not associated with any injury measure in this sample of professional basketball players, suggesting the instrument lacks predictive validity in this population.

## 1. Introduction

Professionals from coaching, healthcare, and scientific circles have long sought to help athletes maximize performance and avoid injury. While methods exist within each profession aimed at predicting performance and lessening the risk of injury, each method possesses inherent limitations regarding predictive validity [1,2]. Thus, no method presently provides a comprehensive measure of either an athlete’s performance capacity or their susceptibility to injury.

The literature suggests that significant rates of overuse or non-contact injuries (NCI) lead to decreased function in athletic populations [3,4,5]. A study by Hreljac et al. estimated that 27 to 70 percent of athletes sustain some type of overuse or NCI [5]. Various authors have attempted to find causal links to such injuries, examining sex [6], biomechanics [7], fatigue [3,8], environmental factors [9], age [10], and other variables, however, the relationships between such factors and risk of injury remain inconclusive.

Consequently, some authors have advocated for more functional or movement-based predictors of injury [5,11,12,13]. These have come in different forms, some targeted particularly at injuries such as the Landing Error Scoring System and anterior cruciate ligaments (ACL) injuries [14,15], and some that are more focused on dynamic postural control such as the Star Excursion Balance Test or the Y Balance Test [16,17,18,19]. Another such model that attempts to predict injury risk is the Functional Movement Screen™ (FMS), which has been promoted as a screening tool that may be used to assess risk of musculoskeletal injury in non-specified athletic populations [20,21]. The FMS is a scored screening instrument that consists of seven movements (deep squat, hurdle step, inline lunge, shoulder mobility, active straight leg raise, trunk stability push-up, and rotary stability) purported to examine movement dysfunctions that identify athletes who are likely more susceptible to injury [20,21]. The FMS model also suggests corrective exercises that may be used to mitigate poor scores on this test battery.

The FMS has received much attention in recent years [22,23,24], and studies have consistently found it to be a reliable instrument [25,26]. However, the scientific evidence for the FMS is less clear regarding its validity for predicting injuries across sports [27,28,29], which is the purported aim of this screening tool. Scientists have invested the FMS in populations ranging from youth sport participants to recreational athletes and across high school, collegiate, and professional athletes [30,31,32,33,34]. However, no studies using the FMS have been published on professional basketball players to date.

Researchers have suggested that the fast and aggressive nature of basketball contributes to injury rates [35,36]. College basketball players appear to have the highest injury prevalence among non-collision sport participants [36]. Starkey [35] suggested that professional basketball players are at increased risk for potential injury due to the longer season, higher number of games, longer game duration, and an increased frequency of games during the week when compared to their collegiate and high school counterparts. Additional data suggest that professional basketball players are two times more likely to experience game-related injuries than collegiate basketball players and that women are injured more often than men [37,38]. Therefore, professional basketball players are an interesting population to study in regard to NCI. The purpose of this study was to investigate the predictive validity of the FMS for measures of NCI in men’s and women’s professional basketball. Based upon previous research, it was hypothesized that professional basketball players demonstrating lower FMS scores captured during pre-season screenings would have a higher association with game-related NCI during National Basketball Association (NBA) or Women’s National Basketball Association (WNBA) season.

## 2. Materials and Methods

This investigation was an observational pilot cohort screening that assessed and scored male and female professional basketball players using the FMS [20,21]. All participants were then tracked during their respective seasons by recording the number of injuries, the anatomical region of the injury, the mechanism of injury, and the time lost due to injury.

### 2.1. Participants

Thirty-two professional National Basketball Association (NBA) and Women’s National Basketball Association (WNBA) players were recruited to participate in the study. The FMS was administered to all invitees at the training camp for the men’s basketball team during September and the women’s basketball team during April. Two participants who ultimately made the open day rosters did not perform the FMS at that time due to pre-existing injuries and were subsequently tested after they received medical clearance to play.

Of the 32 participants (19 men, 13 women) enrolled in this study at the time of training camp, only 27 subjects (15 men, 12 women) were tracked throughout the season due to league-imposed roster limits. Descriptive statistics for the sample are presented in Table 1. Access to the players in this study represented a sample of convenience, limited to the league mandated roster limits for the men’s and women’s franchises willing to participate in this study. All participants gave their informed written consent for inclusion before they participated in the study. The study was conducted in accordance with the Declaration of Helskinki, and the protocol was approved by the university’s Institutional Review Board.

### 2.2. Instruments: The Functional Movement Screen^TM^

The Functional Movement Screen^TM^ (FMS) is a test battery consisting of seven graded components that were assessed using recommended scoring criteria and instrumentation [20,21]. Numerous authors have reported that the FMS has high reliability [22,25,26,39]. While FMS validity studies exist for other athletic populations, no such studies have been published on professional basketball players [27,28,29,39].

### 2.3. Instruments: Tracking Injuries

Several studies have defined an injury as an event (1) occurring due to participation in a practice or a game, (2) requiring examination by the head athletic trainer or other medical staff, and (3) resulting in absence from practice or game for one or more days [36,40,41]. For the present study, Meeuwisse and the co-author’s definition of contact injury (CI) was adopted with one modification: they defined contact injury as the result of direct contact with another player and/or all structures within the basketball arena [36]. For the present study, NCI was operationally defined as any other injury sustained during athletic competition [9,36]. These definitions were clearly explained to the head athletic trainer of each team prior to the study. In addition, the research team reviewed monthly reports that the training staff sent to the league office.

### 2.4. Procedures

The steps in this study included: (1) familiarizing the research team with the FMS and establishing inter-rater reliability, (2) evaluating each participant and assigning an FMS score, (3) collecting weekly data from the head trainer of the men’s and women’s teams, and (4) analyzing all collected data for statistical and clinical merit. The research team practiced implementing the FMS through a pilot study performed on a sample of graduate physical therapy students and then upon members of the summer league team for the men’s NBA basketball team. The pilot results indicated high inter-rater reliability (alpha = 0.91), which is consistent with reliability values previously reported [22,25,26,39].

As noted above, all invitees to training camps for the men’s and women’s professional basketball teams were then evaluated and scored using the FMS [20,21]. Orthopedic clearing tests were also performed, as recommended [20,21]. If the movement needed modification after the first trial and the movement did not cause pain to the participant, two remaining trials were then performed as recommended [20,21]. A final score for each movement was recorded in response to the lowest score (ranging from 0 to 3) achieved during the 3 trials; the lower score was recorded for each of the movements testing right and left sides. Finally, a composite score was calculated for each athlete from the seven movements that comprise the FMS.

Each trial was recorded using 2 digital video cameras (Sony DSC-F707, Sony Corporation of America, New York, NY, USA) placed orthogonally in the cardinal planes, allowing the research team to confirm its inter-rater reliability on the test sample (ICC = 0.97; alpha = 0.97). FMS data were immediately analyzed by the research team; however, FMS scoring was suspended until all data were collected. This was done to lessen the threat to internal validity likely presented by independently grading the men’s and women’s teams four months apart.

For this study, an exposure was defined as the athlete’s participation in any scheduled team physical activity, and these exposures were subsequently coded as a “game”, “practice”, or “shoot around” [36,38,40]. Of the three categories of tracked exposures, games required the highest intensity [35]. Practices occurred on non-game days in the NBA and WNBA, typically running from 1 to 3 h, and were characterized by high intensity effort. The least intense of all three was the “shoot around”, which occurred in the morning before a game. A “shoot around” consisted of relatively light activity, as well as “walking through” the opponent’s strategies. Based upon these three types of exposures, the following variables were tracked: the total number of possible exposures, total number of exposures participated by each athlete, and the total number of exposures missed over the course of either the men’s and women’s seasons.

Data were collected throughout the preseason, regular season, and playoffs for the respective NBA and WNBA seasons. The WNBA season included a one-month hiatus to allow players in the league to participate in the Olympics Games. One WNBA player in this study participated in the Olympic Games and consequently her exposures during this time were not included in this study. (She did not experience any NCI or CI during her Olympic participation.)

In order to maintain player confidentiality, injury updates and daily injury logs were obtained directly from the head athletic trainer of each team and then immediately coded. The code for each player was only known by members of the research team, and the key for the codes used in the study was kept and secured by the primary investigator.

### 2.5. Data Analysis

Descriptive statistics on the participants were calculated using information provided by the head athletic trainer of each team (Table 1). The total number of possible exposures, actual exposures, and missed exposures due to NCI were calculated for each athlete. These data were used to determine crude and specific incident rates for the exposures missed due to NCI per 1000 exposures, and these values where then analyzed based upon the FMS scores demonstrated within the sample.

Crude incident rate (CIR) was defined as the injury rate calculated using the entire sample at risk [42] and thus calculated as the number of exposures missed due to NCI for both the men and women. The specific incident rate (SIR) was defined as the injury rate calculated for the subgroups of the sample [42] and thus calculated as the number of exposures missed due to NCI for participants individually per 1000 exposures. The CIR and SIR were then calculated for the number of NCI per 10 professional basketball players.

FMS scores for each athlete were computed based on the recommended methodology [20,21]. The FMS scores were then used to determine the number of athletes who experienced an NCI versus no NCI based upon the performance on this clinical screen. Then, the statistical sensitivity and specificity of cut scores was calculated on the full distribution of FMS scores.

Then, the ratios were computed for the number of NCIs the athletes sustained relative to the total exposures missed for (1) the individual subgroups and (2) the entire sample of professional basketball players. Ratios were also calculated for the total number of exposures missed due to NCI, CI, and other reasons, respectively [42].

Finally, the FMS data were examined for relationships with descriptive data via one way ANOVA or simple correlations. Data were then analyzed via linear regression models with the FMS score as the predictor and the control variables sex and years of playing experience. The model was assessed for multi-collinearity by calculating variance inflation factor (VIF) statistics. Statistical significance was set a priori at alpha <0.05.

## 3. Results

Twenty-seven participants completed this study (26.31 ± 3.80 year; 192.77 ± 11.70 cm; 90.51 ± 16.67 kg). A sum of 5784 possible exposures existed for the men and women collectively (Table 2). Of the total possible exposures, a sum of 681 exposures was missed, resulting in 5103 actual exposures for all athletes. Of the actual exposures, the men accounted for 3396, missing 564 exposures, whereas the women accounted for the remaining 1707, missing 117 exposures. Table 2 also summarizes the NCI and CI for the exposures missed. When examined as a percentage of total exposures, male players did miss more opportunities (14.4%) than female players (5.9%); however, this result failed to attain significance (F = 3.59, *p* = 0.069).

FMS scores of the participants ranged from 5 to 18 on the 21-point scale for this instrument (Table 3). Of the 27 participants, 19 players (12 men, seven women) sustained an NCI and missed exposures as a result. Men and women did not differ (F = 0.332, *p* = 0.569) in terms of average FMS total scores (male: 15.1, female: 14.4).

Regression models were used to examine the predictive ability of the FMS total score for total exposures missed, NCI exposures missed, and CI exposures missed. The model predicting total exposures missed failed to attain significance (*p* = 0.06) but did predict a significant portion of the variance (r = 0.515). Predictors in the model were sex (Beta = 0.533), FMS total (Beta = −0.017), and years of experience in NBA or WNBA (Beta = −0.284). The model predicting total exposures missed due to NCI similarly failed to attain failed to attain significance (*p* = 0.15) and predict a smaller portion of the variance (r = 0.444). Predictors in the model were sex (Beta = −0.074), FMS total (Beta = 0.182) and years of experience in NBA or WNBA (Beta = 0.467). The regression model for exposures related to CI predicted very little of the variance (r = 0.262) and Beta values for predictors were all below 0.25.

**Figure 1:**
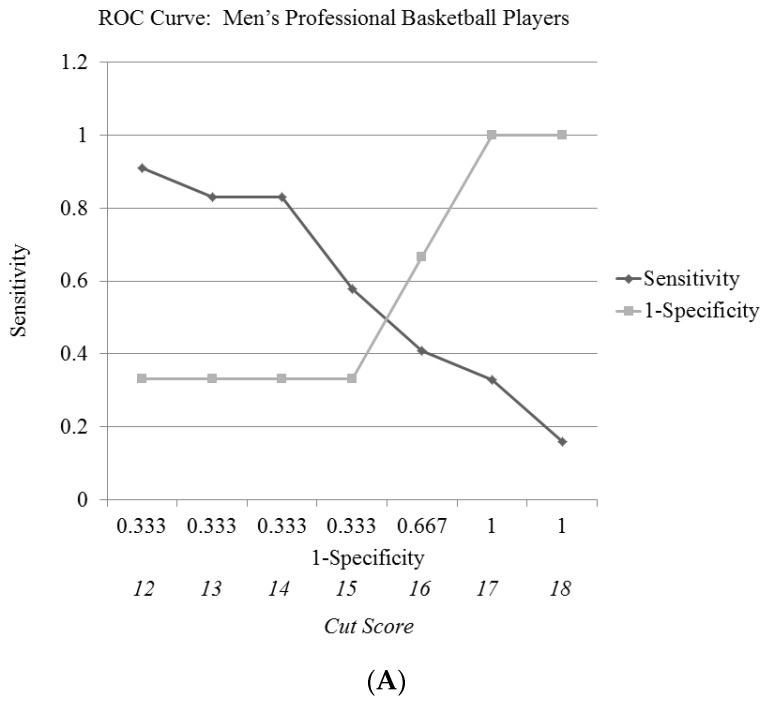

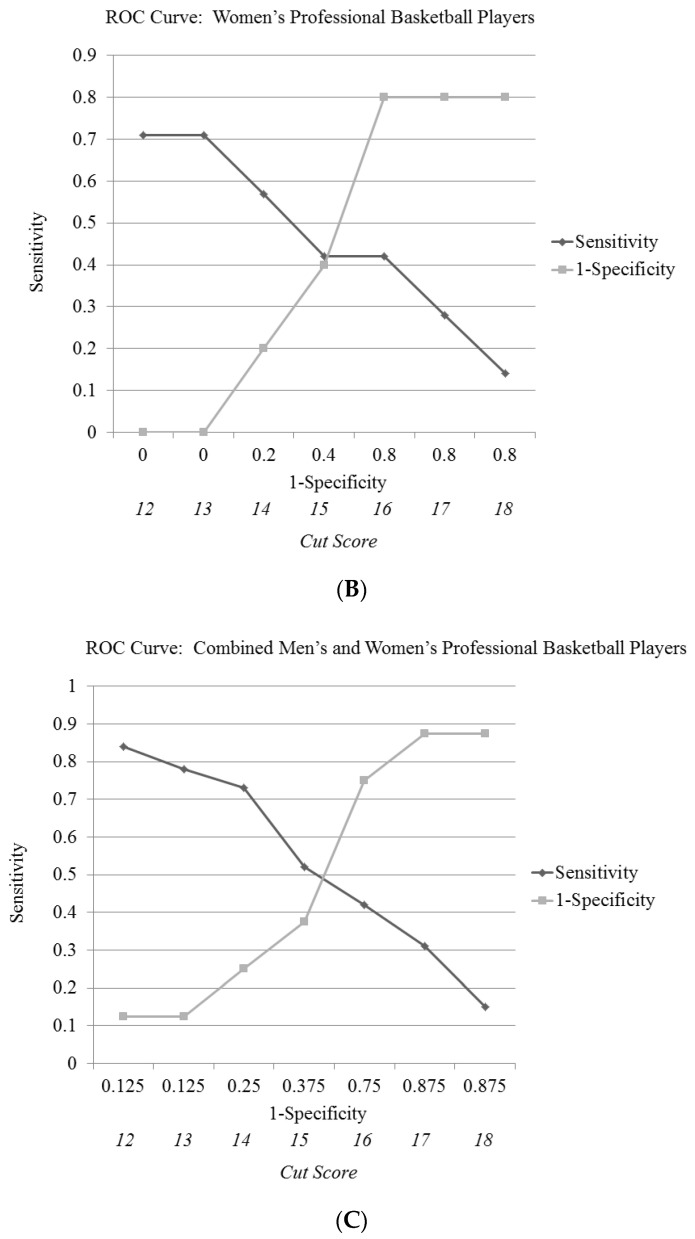
(**A**) Receiver–operating characteristic (ROC) curve of predictive values of the Functional Movement Screen™ (FMS) in men professional basketball players at risk of non-contact injury (NCI). (**B**) Receiver–operating characteristic (ROC) curve of predictive values of the FMS in women professional basketball players at risk of NCI. (**C**) Receiver–operating characteristic (ROC) curve of predictive values of the FMS in combined men and women professional basketball players at risk of NCI.

When the established standard of an FMS score of 14 (at least a two on each section) was used as a binary variable (14 or above, or less than 14) it failed to differentiate the total exposures missed, exposures missed due to NCI or CI via ANOVA (*p* > 0.36). Figure 1 displays the frequency distribution of the FMS composite scores demonstrated among those sustaining an NCI. Similar results were obtained when the FMS total score was transformed to Z-scores and positive and negative values were tested against all missed exposure variables (*p* > 0.50). Separating data by sex and analyzing these same variables (FMS of 14 and Z-score +/-) against all missed exposure variables did not reveal any significant differences (*p* > 0.55).

The 27 athletes in this study experienced a total of 273 missed exposures due to NCI and CI over the course of the men’s and women’s seasons. Of the 273 missed exposures, 160 were due to NCI, while the remaining 113 were due to CI. The ratio of NCI relative to the amount of exposures missed resulted in 32 total injuries, leading to 160 missed exposures. Twelve male participants experienced a total of 22 NCI, resulting in 98 missed exposures and providing a ratio of one injury to 4.5 missed exposures. Seven female participants experienced a total of 10 NCI, resulting in 62 missed exposures and representing a ratio of one injury to 6.2 missed exposures.

## 4. Discussion

The purpose of this observational pilot cohort study was to examine the injury incidence and time lost with a sample of professional basketball players in relation to FMS scores. For the total population in the present study, seven out of ten athletes sustained an NCI and missed 31 out of every 1000 exposures due to NCI. An interesting finding based on these values was the difference demonstrated by male and female participants. The present findings suggested that eight out of ten men will sustain an NCI, while missing 29 of every 1000 exposures due to NCI; conversely, 5.8 of every 10 women will experience an NCI, while missing 36 of every 1000 exposures. These findings suggest that male professional basketball players may be more likely to sustain an NCI than female professional basketball players, although women missed more exposures due to NCI than did men. Literature supports this finding, as several studies have demonstrated that female athletes are susceptible to severe NCI [9,11,43]. Such studies suggest that, for example, women are more likely to experience an anterior cruciate ligament tear NCI than men. The present findings may help to further explain why women athletes typically experience a lesser incidence of NCI but greater missed exposures when compared to men. While some previous studies in other sports have reported relationships between FMS scores and NCI, the present study failed to demonstrate any such relationships in professional basketball players. Additionally, a score of 14 was not found to be a useful predictor [44], nor when the data were standardized with Z-scores did the positive values differ in missed exposures compared to the negative. Overall, in this population of professional athletes, the total FMS score was not related to any form of missed exposures.

There are several possible explanations for the results demonstrated in this study. The FMS has been purported as a means to identify the dysfunctional movement patterns that ultimately contribute to injury [20,21]. While this premise certainly makes sense based upon the existing scientific literature, the battery of tests within this instrument may not possess the specificity necessary for the generalizability to the unique demands professional basketball places upon the musculoskeletal system. The movements included in this test battery are notable for the inclusion of a spectrum of tests that assess strength and dynamic flexibility involving the upper extremities, lower extremities, and trunk—often simultaneously. These seven components address gross movements included in many sporting activities. At the same time, however, the movements included in the test battery are less representative of the often high joint velocities, impacts, impulses, and eccentric loads seen in the human body during vigorous sports such as professional basketball [43,45,46].

Similarly, distribution of FMS scores in this study is somewhat positively skewed, as a majority of the scores in this sample were above the recommended cut score of 14 [44]. In other words, basketball players more likely to experience musculoskeletal breakdown and, in turn, sustain NCI, may be less likely to make it to the professional level in the first place. Similarly, more efficient movement patterns may be among the variables separating the members of this population studied from others who have not made it to this level of play. Repeating this study with a much larger sample of the population of professional basketball players would have obvious scientific value.

A number of other variables may have contributed to the lack of predictive validity of the FMS in this study. The length of the season and total number of exposures are certainly among the variables that may have contributed to these findings. To illustrate, the men in this study played over 100 total games when the preseason, regular season, and playoff contests were tallied, and their season stretched over a period of eight months. The length of the NBA season suggests that professional athletes may certainly experience NCI as a result of residual fatigue in addition to faulty movement patterns or that professional basketball players may experience NCI due to an interaction of these factors. The types of NCIs professional basketball players sustain are dependent upon the physical demands of the game. Professional basketball players may play 40 or more minutes per game, travel the length of a 28.65 m court 200 to 300 times per game, and regularly do so while cutting, jumping, and contacting the opponent [35]. Authors have suggested that the above demands, in addition to the stress associated with traveling, may predispose professional basketball players to the development of acute and NCI [35]. Moreover, a commonly held belief within the NBA is that games on successive nights, or “back to backs”, often in different cities with the associated travel, serve to further undermine the capacity of basketball players in this league to recuperate from the physical demands placed upon their bodies. In short, such cycles may predispose professional basketball players to increased injury and may account for the higher injury incidence we found in the present study [35,37,38,47].

Previous injury history and the unique anthropometric features of professional basketball players are other variables that may have contributed to the lack of predictive validity of the FMS in this study. At least one study makes a case for the importance of examining past medical history and previous injury when attempting to examine injury mechanism and incidence [36]. Several studies have also identified past injury as a positive risk factor for identifying the rates and risks of injury for intercollegiate basketball players [36,48]. Individuals with faulty movement patterns may have a history of previous injury, but the causal relationship between movement patterns and previous injury remains unclear. A large number of the athletes in this study had past injuries that may have contributed to both the NCIs and CIs seen throughout the season. The FMS does not account for any past injuries in its predictive estimate. Contributing to the difficulty in ascertaining the value of the FMS as a predictive instrument is the observation that this instrument likely does not solely test the mobility and stability that may contribute to NCI. Krackow [29] found that the taller, heavier, and higher relative body weight football players achieved lower FMS scores than shorter, lighter, and lower BMI football players. These findings suggest that lower FMS might not be a result of insufficient mobility and stability, but rather the FMS may possess an inherent bias in atypical athletic populations [29]. This issue raises obvious concerns in this study given the anthropometric characteristics displayed by the typical NBA or WNBA player. Further study is needed to assess the validity of this instrument in athletic populations with such atypical anthropometric characteristics compared to a general athletic population.

The methodological approach of executing this study by combining the men’s and women’s team as a means of increasing the sample size has limitations. Female athletes have been identified as having additional risk factors for injury, as well as being more likely to sustain a more serious injury than male athletes [9,11]. Additional risk factors for female athletes noted in the scientific literature include increased joint laxity, decreased strength, anatomical differences (tibial width, femoral notch width, condylar notch), height to weight ratios, and different landing strategies when compared to men [9,45]. Such differences may have affected the present findings and repeating this study with larger samples of both men and women may help to shed light on injury differences based on sex.

The FMS has generated increasing interest in sports medicine for its potential in identifying individuals who move poorly and who may be at risk of sustaining an injury during athletic participation. Such instruments need scientific scrutiny if their potential is to be realized and they are to be used on a widespread basis. This study represents an important step toward such a goal.

It is important to acknowledge the limitations of the current study, as there are some factors to consider that may have affected the results of the present study. Potential limitations of this study center on the available study population. This study was based on a sample of convenience to a unique, professional athletic population, nonetheless the study sample size may not have provided the necessary statistical power to determine a cut score on the FMS with high sensitivity and specificity. A larger sample size, such as that which might be accomplished through implementing this study on a league-wide basis, would provide greater statistical power. Additionally, we chose to use pre-existing definitions of NCI defined in the literature. Therefore, our definition may have excluded some incidents of NCI, as some participants did experience an NCI but did not miss any exposures so thus were not coded as injured. Finally, there were variables that were not controlled in this investigation, such as the number of minutes played per game, player position, amount of cross training, and preferred basketball shoe. Though these variables were not controlled, these findings may be reasonably generalized to clinical scenarios where these variables are also not typically controlled. Future studies of the predictive validity of the FMS within professional basketball players should have a larger sample size and might control the number of minutes played per game, player position, amount of cross training, and preferred basketball shoe to more fully explore the validity of this screen.

## 5. Conclusions

Based on the present findings, the demonstrated FMS scores lacked predictive validity for musculoskeletal injuries within this sample of professional basketball players. Neither the recommended cut score of 14 nor other cut scores showed a strong balance between sensitivity and specificity. This study also documented that men sustained more NCIs than women, but the women experienced greater time loss as a result of such injuries. Further study is necessary to determine whether the FMS has predictive validity in this and other athletic populations, as well as to better describe the lost time characteristics among professional basketball players.

## Figures and Tables

**Table 1:** Descriptive statistics for participant professional basketball players.

Group	Age (Year)	Height (cm)	Weight (kg)	BMI	Years in League	FMS
Men	26.31 ± 3.80 (range: 21–38.13)	200.2 ± 9.71 (range: 185.42–213.36)	101.70 ± 12.50 (range: 83–123.37)	25.31 ± 1.94 (range: 21.9–28.6)	5.13 ± 4.21 (range: R to 16)	15.1 ± 2.2 (range: 11–18)
Women	25.92 ± 3.20 (range: 21–33)	183.72 ± 7.21 (range: 170.18–193.04)	77.12 ± 10.69 (range: 63.64–95.45)	22.7 ± 2.26 (range: 20–26.9)	3 ± 1.54 (range: R to 5)	14.4 ± 3.60 (range: 5–18)
Combined	26.31 ± 3.80 (range: 21–38)	192.77 ± 11.70 (range: 170.20–213.36)	90.51 ± 16.67 (range: 63.63–123.37)	24.11 ± 2.40 (range: 20–28.6)	4.04 ± 3.45 (range: R to 16)	14.75 ± 2.87 (range: 5–18)

R = rookie.

**Table 2:** Exposure data and incident rates for missed exposures.

Value	Men	Women	Total
Total Possible Exposures	3960	1824	5784
Total Actual Exposures	3396	1707	5103
Total Missed Exposures	564	117	681
Exposures Missed Due to NCI	98	62	160
Exposures Missed Due to CI	78	35	113
Exposures Missed Due to Other	388	20	408
Exposures Missed Due to NCI per 1000 Actual Exposures	29	36	33

NCI—non-contact injury; CI—contact injury.

**Table 3:** FMS score summary relative to athletes sustaining NCI versus no NCI based on the FMS score.

	Male		Female		Total	
FMS Score	NCI	No NCI	NCI	No NCI	NCI	No NCI
5	0	0	1	0	1	0
11	1	0	1	0	2	0
12	1	1	0	0	1	1
13	0	0	1	0	1	0
14	3	0	1	1	4	1
15	2	0	0	1	2	1
16	1	1	1	2	2	3
17	2	1	1	0	3	1
18	2	0	1	1	3	1
Total	12	3	7	5	19	8
